# Clinical Application of Micronucleus Test: A Case-Control Study on the Prediction of Breast Cancer Risk/Susceptibility

**DOI:** 10.1371/journal.pone.0112354

**Published:** 2014-11-21

**Authors:** Claudia Bolognesi, Paolo Bruzzi, Viviana Gismondi, Samantha Volpi, Valeria Viassolo, Simona Pedemonte, Liliana Varesco

**Affiliations:** 1 Unit of Environmental Carcinogenesis, IRCCS AUO San Martino-IST Istituto Nazionale Ricerca sul Cancro, Genoa, Italy; 2 Unit of Clinical Epidemiology, IRCCS San Martino-IST Istituto Nazionale Ricerca sul Cancro, Genoa, Italy; 3 Unit of Hereditary Cancer, IRCCS San Martino-IST Istituto Nazionale Ricerca sul Cancro, Genoa, Italy; National Research Council, Italy

## Abstract

The micronucleus test is a well-established DNA damage assay in human monitoring. The test was proposed as a promising marker of cancer risk/susceptibility mainly on the basis of studies on breast cancer. Our recent meta-analysis showed that the association between micronuclei frequency, either at baseline or after irradiation, and breast cancer risk or susceptibility, has been evaluated in few studies of small size, with inconsistent results. The aim of the present study is to investigate the role of micronucleus assay in evaluating individual breast cancer susceptibility. Two-hundred and twenty untreated breast cancer patients and 295 female controls were enrolled in the study. All women were characterized for cancer family history and 155 subjects were evaluated for the presence of BRCA mutations. Micronuclei frequency was evaluated at baseline and after irradiation with 1-Gy gamma rays from a 137Cs source. The results show a non significant increase of frequency of micronucleated binucleated lymphocytes in cancer patients compared with the controls at baseline (Mean (S.E.): 16.8 (0.7) vs 15.7 (0.5), but not after irradiation (Mean (S.E.): 145.8 (3.0) vs 154.0 (2.6)). Neither a family history of breast cancer nor the presence of a pathogenic mutation in BRCA1/2 genes were associated with an increased micronuclei frequency. Our results do not support a significant role of micronucleus frequency as a biomarker of breast cancer risk/susceptibility.

## Introduction

The cytokinesis-block micronucleus assay (CBMN assay), due to its ability to detect both structural and numerical chromosomal aberrations, is one of the most successful assays in genetic toxicology and mutation research.

In vitro, it is recommended in the basic battery of tests to screen new chemical and physical agents for genotoxicity [1]. However, it is its use in humans that generated most interest, with three possible purposes:

- assessment of the exposure to genotoxic agents
- evaluation of individual susceptibility to the effects of exogenous or endogenous genotoxic agents
- assessment of the risk of developing cancer and other chronic diseases, as a marker of “early damage”.

In this perspective, CBMN could represent a formidable tool for epidemiological studies. Indeed, the CBMN assay is a well-established method to assess recent exposure of individuals to genotoxic agents in humans, with multiple applications, the most important and validated one being evaluation of exposure to ionizing radiations [2]. Its use in evaluating the individual susceptibility to potentially genotoxic exposures could be even more important for clinical and epidemiological studies and for preventive interventions, because CBMN could be used to identify individuals at high risk of developing a given disease, and could even qualify as an intermediate biomarker (surrogate endpoint) to assess the effects of preventive measures. However, the overall evidence from studies aimed at assessing these two potential applications is inconclusive.

In prospective studies evaluating large cohorts of disease-free subjects, an increase in micronuclei (MN) frequency in peripheral blood lymphocytes was associated with an increased cancer risk at the population level, providing suggestive evidence that this biomarker may be predictive of cancer risk [3], [4]. Many studies were also published on the application of the MN test in peripheral lymphocytes in untreated patients with cancer or preneoplastic lesions, the large majority of them showing a significant increase of MN frequency in patients compared to control groups [5]–[10]. Increased MN frequencies were also reported in a number of studies in patients with neurodegenerative diseases [11], cardiovascular diseases and diabetes [12]. Moreover, an increased MN frequency was detected in subjects affected by cancer-associated congenital syndromes characterized by a deficiency in DNA damage response [13], [14].

The large number of diseases and conditions found to be associated with an increase in MN frequency raises a first problem: if all or most of these associations are true, CBMN would lack the specificity which is needed for clinical applications; otherwise, if most of these association are spurious, a close scrutiny of the methodological quality of these studies could reveal it and indeed, most of these studies were of small size, with potentially biased selection of cases and controls, and with inadequate control of potential confounding factors, including therapies and diagnostic tests.

A second problem is related to the moderate differences in MN frequency between cases and controls [5]–[10], or, in cohort studies, to the lack of a clear dose-response relationship [3]. The modest strength of the observed associations does not allow to rule out that they are due to unrecognized bias or to confounding factors.

The third problem is the large variability in CBMN frequency: this variability is observed within individuals, within the same laboratory across individuals or in different periods, and across different laboratories. The results of the analysis of pooled data reveal a large variability among the labs: this variability was largely attributable to known factors, including, beside genotoxic factors and host factors, methods and scoring criteria with less than 25% of the observed variability unexplained [15], [16]. Conversely, many of the determining factors of the individual variation in background levels of MN in lymphocytes are still unknown, although the sensitivity of the CBMN assay to multiple endogenous and exogenous genotoxic factors, individual genetic susceptibility and ageing, may do play a role [17].

The variability in the results of CBMN assays observed in different studies and laboratories is such that no range of “normal values” has ever been proposed: even among supposedly healthy controls unexposed to genotoxic agents, a>4-fold variation in average MN frequencies was observed in different studies [15]. Notwithstanding all these limitations, the hypothesis that CBMN assay can be used for preventive purposes is still considered very promising, and has been and still is pursued in a large number of epidemiological and clinical studies.

Many of these studies were on breast cancer (BC), partly based on the hypothesis that CBMN could help identifying those women with an inherited predisposition. This hypothesis has a strong biological rationale, since both genes known to cause hereditary BC, (BRCA1 and BRCA2) are known to be associated with a defective DNA repair [18]–[20] However, the evidence in support of this hypothesis is rather weak and based on small studies of questionable methodological quality [21]–[23].

Based on these premises, in 2009 we started the present case-control study with the primary aim of evaluating if the presence of a pathogenic mutation in either or both the BRCA genes is associated with an increased MN frequency. If confirmed, this association could have two practical uses for MN assay: a) as a prescreening tool, to select women for genetic tests; and b) as a functional test, to help interpreting BRCA variants of unknown significance (VUS), which represent a major problem in genetic counseling for women with familiar BC.

Mutagen sensitivity, measured by quantifying the genotoxic events induced by in vitro irradiation to gamma rays of peripheral blood lymphocytes, was used with the aim to improve the detection of the individual genetic susceptibility.

## Material and Methods

### Study subjects

The subjects in this study were recruited in our Institute among: a) patients and their relatives referring for a genetic consultation to the Hereditary Cancer Center; b) women attending the Radiology Unit for mammography and c) BC cases attending the Oncology Department for follow up. All women aged 18–80 years, with and without BC, consenting to provide a blood sample and information on their BC family history, were considered eligible for this study. Women with other cancers or with known or suspected hereditary cancer syndromes other than Hereditary Breast Ovarian Cancer were excluded. BC patients were enrolled only if they had not received chemo- or radio-therapy within 12 months before blood sampling.

The obvious bias deriving from the setting where cases and controls were selected affects the relative frequency of women with a positive family history of BC and/or a BRCA mutation, but does not affect the endpoint of our study, that is, the MN frequency in these different groups of women.

**Figure 1:**
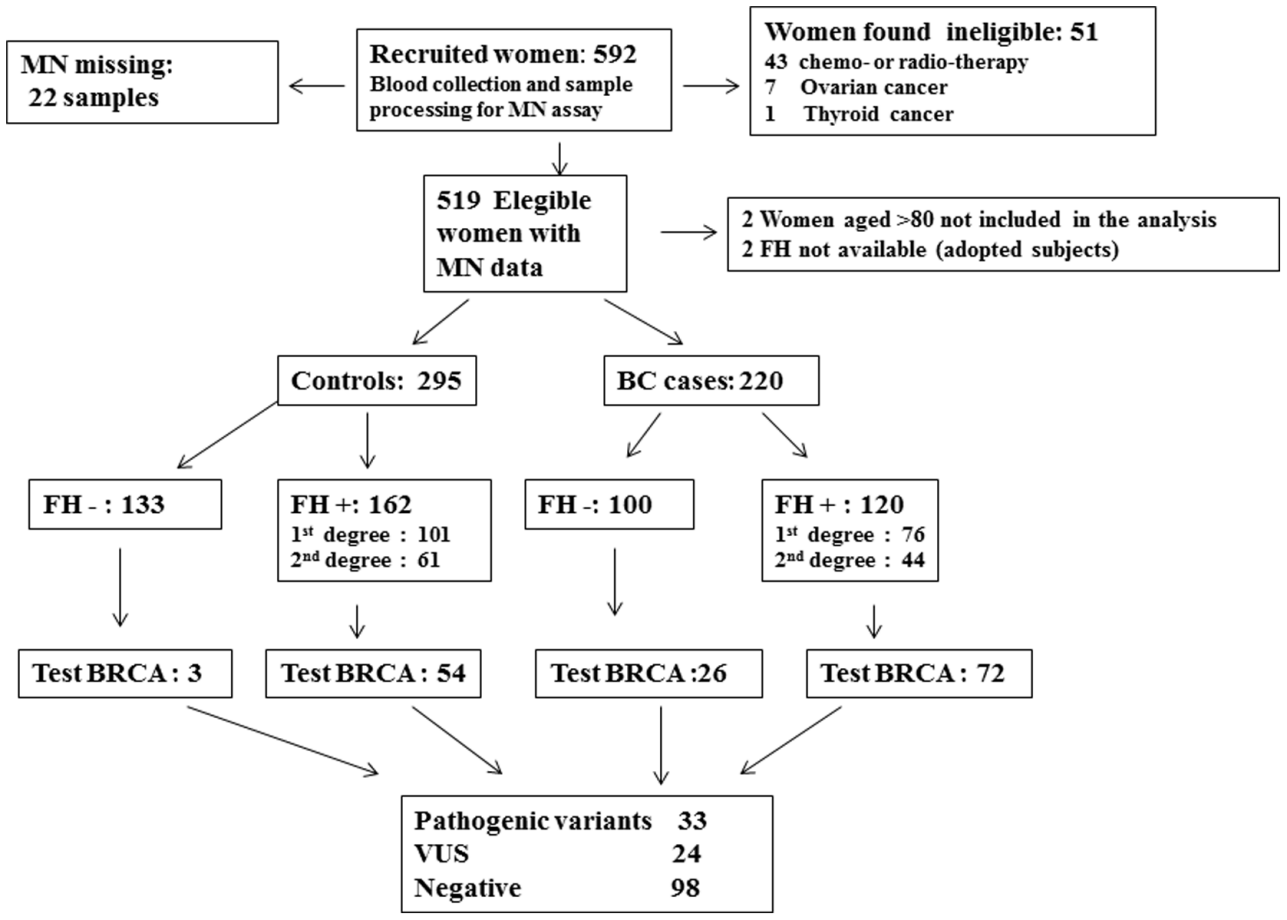
Flow diagram for the selection of study subjects.

The details of the study population are described in the flow chart (fig 1). The entire study population includes 592 women recruited in seven years (January 2006-May 2012). Among the recruited individuals there were 51 ineligible subjects: 43 cases had received radio or chemotherapy less than 12 months before sampling, and 8 subjects were affected only by other tumours. Furthermore, in 22 subjects MN results were missing, leaving 519 eligible subjects with MN data. Finally, two subjects who could not report their cancer family history because they had been adopted and 2 women aged more than 80 were excluded. Therefore, the analysis includes 515 subjects: 295 controls and 220 BC patients. All women in the study were informed on the aims and methods of the study and gave a written consent to the use for study purposes of the clinical and family history information and to provide a blood sample for MN test, which was drawn after provision of the written consent.

Detailed cancer family history information was collected by means of a personal interview with pedigree reconstruction up to three generations on both sides of the family.

In all analyses, only women with BC in a 1^st^ or 2^nd^ degree relative were considered to have a positive family history.

During the study period, BRCA genetic testing was proposed to women according to protocols currently in use in Hereditary Cancer Center, and no test was proposed as a consequence of participation to the study. Accordingly, the results of BRCA tests were abstracted from clinical records. BRCA variant classification followed international rules [24] and variants were distinguished in three classes: - pathogenic; - uncertain significance (VUS); - not pathogenic or of no clinical significance.

### Ethics statement

All research was carried out in compliance with the Helsinki Declaration. The protocol of the study and the consent procedure were approved by the local Ethical Committee of the National Cancer Research Institute of Genoa (N. ECE08.001, 07/04/08). From all participants, informed written consent to the study was obtained and recorded.

### Micronucleus test

Heparinized blood samples, sent to the lab within 24 h, were used for the establishment of the lymphocyte cultures. Each blood sample (2,5 ml) was divided into two parts: one was used for mutagen sensitivity assay and exposed at 0°C to 1-Gy gamma ray from a 137 Cs source at a dose rate of 0.15 Gy/sec (7 sec) (irradiated sample), the other was not irradiated (baseline sample).

The modified cytokinesis-blocked method was used to determine frequency of micronuclei (MN) [25]. Three sterile whole blood cultures for both samples were prepared. A 0.3 ml aliquot of whole blood was incubated at 37°C in 4.7 ml of RPMI 1640 (Life Technologies, Milano, Italy) supplemented with 10% fetal bovine serum (Gibco BRL, Life Technologies SrL, Milano, Italy), 1.5% phytohemoagglutinin (Murex Biotech, Dartford, UK), 100 Unit/ml penicillin and 100 µg/ml streptomycin. After 44 h, Cytochalasin B (Sigma, Milano, Italy) was added at a concentration of 6 µg/ml. At the end of incubation at 37°C for 72 h, cells were centrifuged (1000 rpm, 10 min) then treated with 5 ml of 0.075 mM KCl for 3 min at room temperature to lyse erythrocytes. The samples were then treated with prefixative (methanol: acetic acid 3∶1) and centrifuged. The cellular pellets were resuspended in 5 ml of methanol, then centrifuged. Treatment with fixative (methanol: acetic acid 5∶1) followed by centrifugation was repeated twice for 20 min. Lymphocytes in fresh fixative were dropped onto clean iced slides, air-dried and stained in 2% Giemsa (Sigma, Milano, Italy). MN analysis was performed blind only on lymphocytes with preserved cytoplasm. Two thousand binucleated (BN) cells were analyzed for each subject. Cells were cytologically scored using the cytome approach to evaluate viability status (necrosis, apoptosis), mitotic status (mononucleated, binucleated, multinucleated) and chromosomal damage or instability status (presence of micronuclei, nucleoplasmic bridges, nucleoplasmic buds) [25]. The cytokinesis-block proliferation index (CBPI) was calculated as: CBPI  =  (M1 + 2M2 + 3M3)/N where M1–M3 represent the numbers of cells with 1–3 or more nuclei and N is the total number of viable cells scored (excluding necrotic and apoptotic cells).

The results are reported as MNBN/1000 BN cells.

### Statistical analyses

In most univariate analyses, the distribution of MN values in different groups of patients was compared by means of the Mann- Whitney non parametric test. However, in order to take into account the skewness of the distributions of MN frequencies in different subjects, the logarithmic transformation of MN frequencies was used in multivariate linear regression analyses, where the distributions of MN frequencies in different groups of women were compared while adjusting for potential confounding variables. Potential confounders considered in these analyses were variables that in univariate analyses appeared to be associated with MN frequency, and include age, menopausal status, family history of BC, number of children and the year of sample collection/analysis. All tests are 2-sided. SPSS. Statistics 20.0 was used in all statistical analyses.

## Results

### Characteristics of the study populations

Cases were older than controls (median age 56, range 31–79 and median age 47, range 18–72 years, respectively) and more often post menopausal (79% vs 39%) (Table 1). There were no significant case–control differences in the distributions of body mass index, age of menarche, number of children, smoking habits, total number of children and use of hormone replacement therapy. An older age at first pregnancy and a more frequent use of Oral Contraceptives were observed in controls than in BC cases. Due to the recruitment of a large number of subjects in the Hereditary Cancer Center, the proportion of women with a positive 1^st^/2^nd^ degree family history of BC was high and not significantly different in BC cases and controls (fig.1). A higher proportion of BC cases than controls was evaluated for the presence of BRCA mutations (98/220, 44.5% vs 57/295, 19.3%, fig.1).

**Table 1:** Characteristics of Study Subjects.

	BC Cases No (%)	Controls No (%)	Total No (%)
Total	220 (100)	295 (100)	515 (100)
Age			
mean (range)	56 (31–79)	47 (18–72)	51 (18–79)
<40	16 (7.3)	66 (22.3)	82 (15.9)
40–55	80 (36.3)	160 (54.2)	240(46.6)
55–65	76 (34.5)	51 (17.3)	127 (24.7)
>65	48 (21.8)	18 (6.1)	66 (12.8)
Weight (N/average/SE)#	183/63.48/0864	266/61.96/0.654	449/62.58/0.524
Height (N/average/SE) #	184/161.52/0.459	266/162.53/0.362	450/162.12/0.286
BMI			
<20	23 (12.6)	47 (17.8)	70 (15.7)
20–24	97 (53.3)	141 (53.4)	238 (53.4)
25–29	42 (23.1)	62 (23.5)	104 (23.3)
30+	20 (11.0)	14 (5.3)	34 (7.6)
NA	38	31	69
Age at menarche #			
<12	44 (23.9)	70 (26.3)	114 (25.3)
12	52 (28.3)	76 (28.6)	128 (28.4)
13+	88 (47.8)	120 (45.1)	208 (46.2)
Age at 1st pregnancy			
Nulliparous	48 (21.8)	72 (24.4)	120 (23.3)
<25	54 (24.5)	55 (18.6)	109 (21.2)
25–30	67 (30.5)	77 (26.1)	144 (28.2)
>30	51 (23.2)	91 (30.8)	142 (27.6)
N.of Children			
Nulliparous	48 (21.8)	72 (24.4)	120 (23.3)
1	83 (37.7)	100 (33.9)	183 (35.5)
2	73 (33.2)	104 (35.3)	177 (34.4)
3+	16 (7.3)	19 (6.4)	35 (6.8)
Menopausal status*			
Premenopausal	46 (20.9)	179 (60.7)	225 (43.7)
Postmenopausal	174 (79.1)	116 (39.3)	290 (56.3)
Smoking Status			
Never	106 (57.9)	145 (55.5)	251 (56.6)
Current	35 (19.1)	61 (23.4)	96 (21.6)
Ex smoker	42 (23.0)	55 (21.1)	97 (21.8)
NA	37	34	71
Oral Contraceptive use			
Ever	102 (46.4)	174 (59.0)	276 (53.6)
Never	83 (37.7)	92 (31.2)	175 (34.0)
NA	35 (15.9)	29 (9.8)	64 (12.4)
Hormone Replacement therapy			
Ever	24 (10.9)	38 (12.9)	62 (12.0)
Never	159 (72.3)	224 (75.9)	383 (74.4)
Unknown	37 (16.8)	33 (11.2)	70 (13.6)

S.E. standard error.

NA not available.

# Information on weight and height, age at menarche and smoking status was missing for 64–71 cases.

* 57 women whose menopausal status was unknown were classified as premenopausal if age <50 and postmenopausal if age ≥50.

### Frequency of binucleated cells with micronuclei by case-control status

MN frequency at baseline and after in vitro irradiation at 1 Gy by case-control status in the total study population and in subgroups is shown in table 2.

**Table 2:** Effect of covariates on frequency of binucleated cells (BN) with micronuclei (MNBN/1000BN) BN) at baseline and after in vitro irradiation at 1 Gy by case-control status.

	BC Cases			Controls		
	N	MNBN/1000BN mean (S.E.)		N	MNBN/1000BN mean (S.E.)	
		Baseline	1Gy		Baseline	1 Gy
Overall	220	16.8 (0.7)	145.8 (3.0)	295	15.7 (0.5)	154.0 (2.6)
P value		0.201	0.046			
Age						
<40	16	10.7 (1.4)	135.4 (8.6)	66	12.2 (1.0)	139.3 (5.5)
40–55	80	15.6 (1.0)	143.3 (5.2)	160	15.3 (0.6)	157.5 (3.5)
55–65	76	19.3 (1.6)	145.9 (5.1)	51	20.2 (1.1)	162,6 (6.4)
>65	48	17.1 (1.2)	153.4 (7.1)	18	20.2 (2.3)	152.1 (11.5)
P value		0.016	0.501		<0.001	0.022
BMI						
<20	23	16.9 (2.1)	150.6 (10.1)	47	14.0 (1.2)	160.3 (7.1)
20–24	97	17.4 (1.3)	154.0 (4.4)	141	16.2 (0.7)	157.1 (3.3)
25–29	42	18.2 (1.4)	159.4 (6.2)	62	18.1 (0.994)	167.0 (6.1)
30+	20	16.4 (1.7)	160.5 (5.8)	14	17.8 (2.085)	157.8 (9.0)
P value		0.946	0.782		0.075	0.516
Age at menarche						
<12	44	19.5 (2.4)	159.3 (5.8)	70	16.5(1.0)	162.4 (4.7)
12	52	17.4 (1.5)	151.1 (6.0)	76	15.1 (0.8)	154.9 (4.9)
13+	88	16.2 (0.9)	155.0 (4.5)	120	17.0 (0.8)	161.8 (4.1)
P value		0.285	0.637		0.313	0.472
Age at 1st pregnancy						
Nulliparous	48	17.3 (2.3)	147.4 (6.7)	72	14.4 (1.1)	148.3 (5.4)
<25	54	17.3 (1.3)	143.0 (6.5)	55	17.5 (1.2)	158.7 (6.4)
25–30	67	16.8 (1.1)	151.6 (5.7)	77	16.8 (0.9)	154.9 (5.4)
>30	51	15.9 (1.2)	139.6 (6.0)	91	14.9 (0.8)	154.8(4.5)
P value		0.906	0.520		0.094	0.626
N. of Children						
Nulliparous	48	17.3 (2.3)	147.4 (6.7)	72	14.4 (1.1)	148.3 (5.4)
1	83	16.0 (0.9)	143.9 (5.2)	100	15.1 (0.7)	154.5 (4.5)
2	73	16.9 (1.0)	148.5 (5.5)	104	16.9 (0.8)	157.2 (4.6)
3+	16	19.7 (2.5)	138.4 (7.7)	19	18.0 (2.1)	155.2 (10.3)
P value		0.631	0.837		0.109	0.653
Menopausal status						
Premenopausal	46	14.6 (1.3)	136.1 (6.9)	179	14.1 (0.6)	150.2 (3.4)
Postmenopausal	174	17.4 (0.8)	148.4 (3.4)	116	18.3 (0.8)	159.9 (4.2)
P value		0.111	0.108		<0.001	0.075
Smoking Status						
Never	106	17.3 (0.8)	157.0 (3.9)	145	16.4 (0.7)	160.1 (3.5)
Current	35	18.1 (2.0)	152.6 (7.8)	61	15.2 (1.1)	156.7 (5.8)
Ex smoker	42	17.1 (2.5)	152.3 (6.7)	55	17.5 (1.1)	164.8 (6.1)
P value		0.908	0.774		0.343	0.598
Oral Contraceptive use						
Ever	102	18.2 (1.3)	157.2 (4.2)	174	15.7 (0.6)	161.3 (3.2)
Never	83	16.2 (1.0)	151.3 (4.6)	92	17.5 (1.0)	157.5 (4.7)
P value		0.229	0.348		0.107	0.495
Hormone Replacement therapy						
Ever	24	20.0 (2.0)	157.5 (7.6)	38	18.0 (1.4)	153.0 (5.9)
Never	159	16.9 (0.9)	154.6 (3.4)	224	16.0 (0.6)	161.7 (3.0)
P value		0.215	0.751		0.195	0.253

S.E. standard error.

Overall, no significant difference was seen between cases and controls, although a higher frequency was observed at baseline in cases as compared to controls (Mean (S.E.): 16.8(0.7) vs 15.7 (0.5) while the opposite was true after irradiation (Mean (S.E.): 145.8(3.0) vs 154.0 (2.6)). An age-related increase of baseline MNBN/1000BN was observed in BC cases and in controls (p = 0.016 and p<.001 respectively), while the age effect was less evident after in vitro irradiation (p = 0.501 and p = 0.022, respectively). The association with age was reflected in the analysis according to menopausal status, particularly among controls. No clear effect of BMI, age at menarche, age of first pregnancy, number of children, smoking habits, oral contraceptive use and hormone replacement therapy on MNBN frequency was seen, either without or after irradiation, and the patterns were similar in cases and controls.

The mean CBPI value of the whole population at baseline was 1,65 (range 1.08–2.30). A negative correlation between PI and age was observed in both cases and control groups. No statistically significant difference was detected for any considered covariate. A decrease in PI value was observed after in vitro irradiation (mean value 1,54) without any difference by case-control status.

Data obtained on other nuclear anomalies (nuclear buds (NB) and nuclear plasmatic bridges (NPB)) in binucleated cells and on frequency of micronuclei in mononuclear lymphocytes (MNMONO), at baseline and after in vitro irradiation at 1 Gy didn't show any statistically significant difference for any considered covariate.

### MN frequency according to Family History of Breast cancer (BC FH) and BRCA status

The prevalence of pathogenic variants detected among those assessed was similar in the two groups (20/98, 20% and 13/57, 23%, respectively) (table 3). No strong evidence in support of an association between the presence and type of BC FH and MN frequency at baseline and after irradiation was seen. Among cases, baseline MNBN/1000 BN lymphocytes was 16.9, 18.5 and 16.0 in those with 1^st^ degree, 2^nd^ degree, and>2^nd^/No family history of BC, whereas, after irradiation, the corresponding figures were 136.6, 148.0 and 151.8. Among controls, a statistically significant association between BC FH and MNBN/1000 BN lymphocytes was seen both at baseline and after irradiation (p = 0.033 and p = 0.006, respectively). However, the observed trend was the opposite of the expected one, with the highest values in the group with>2^nd^ degree or negative BC FH: observed values were 14.6, 14.6 and 17.1 at baseline in those with a 1^st^ degree, 2^nd^ degree and no/>2^nd^ degree BC FH, respectively while the corresponding values after irradiation were 142.5, 157.4, 161.1.

**Table 3:** Effect of Cancer Family History and BRCA status on frequency of binucleated cells (BN) with micronuclei (MNBN/1000BN) at baseline and after in vitro irradiation at 1 Gy by case-control status.

	BC Cases			Controls			Total		
	N	MNBN/1000BN Mean(S.E.)		N	MNBN/1000BN Mean(S.E.)		N	MNBN/1000BN Mean(S.E.)	
		Baseline	1Gy		Baseline	1 Gy		Baseline	1 Gy
**Family history**									
1st degree	76	16.9 (1.5)	136.6 (5.5)	101	14.6 (0.8)	142.5(4.2)	177	15.6 (0.8)	140.0 (3.4)
2nd	44	18.5 (1.7)	148.0 (7.0)	61	14.6 (1.0)	157.4(6.3)	105	16.2 (0.9)	153.5 (4.7)
Neg/>2nd	100	16.0 (0.8)	151.8(4.3)	133	17.1 (0.7)	161.1(3.9)	233	16.7 (0.6)	157.1 (2.9)
P value		0.448	0.089		0.033	0.006		0.519	0.001
**BRCA classes**									
BRCA1	14	12.9 (1.7)	138.2 (12.7)	10	9.7 (2.4)	112.8 (14.2)	24	11.6 (1.4)	127.6 (9.6)
BRCA2	6	22.3 (6.8)	147.6 (19.5)	3	7.2 (0.3)	100.3 (23.1)	9	17.3 (5.0)	131.8 (16.3)
VUS BRCA1	6	13.7 (2.1)	122.9 (16.6)	2	9.25 (1.7)	97.2 (19.5)	8	12.6 (1.7)	116.5 (13.4)
VUS BRCA2	11	14.0 (2.9)	116.6 (13.0)	5	20.4 (5.4)	141.8 (29.9)	16	16.0 (2.6)	124.5 (12.7)
Negative test	61	15.9 (1.2)	129.7 (6.8)	37	11.2 (1.1)	122.2 (6.9)	98	14.1 (0.9)	126.9 (5.1)
P value		0.311	0.741		0.061	0.621		0.411	0.974

S.E. standard error.

**Figure 2:**
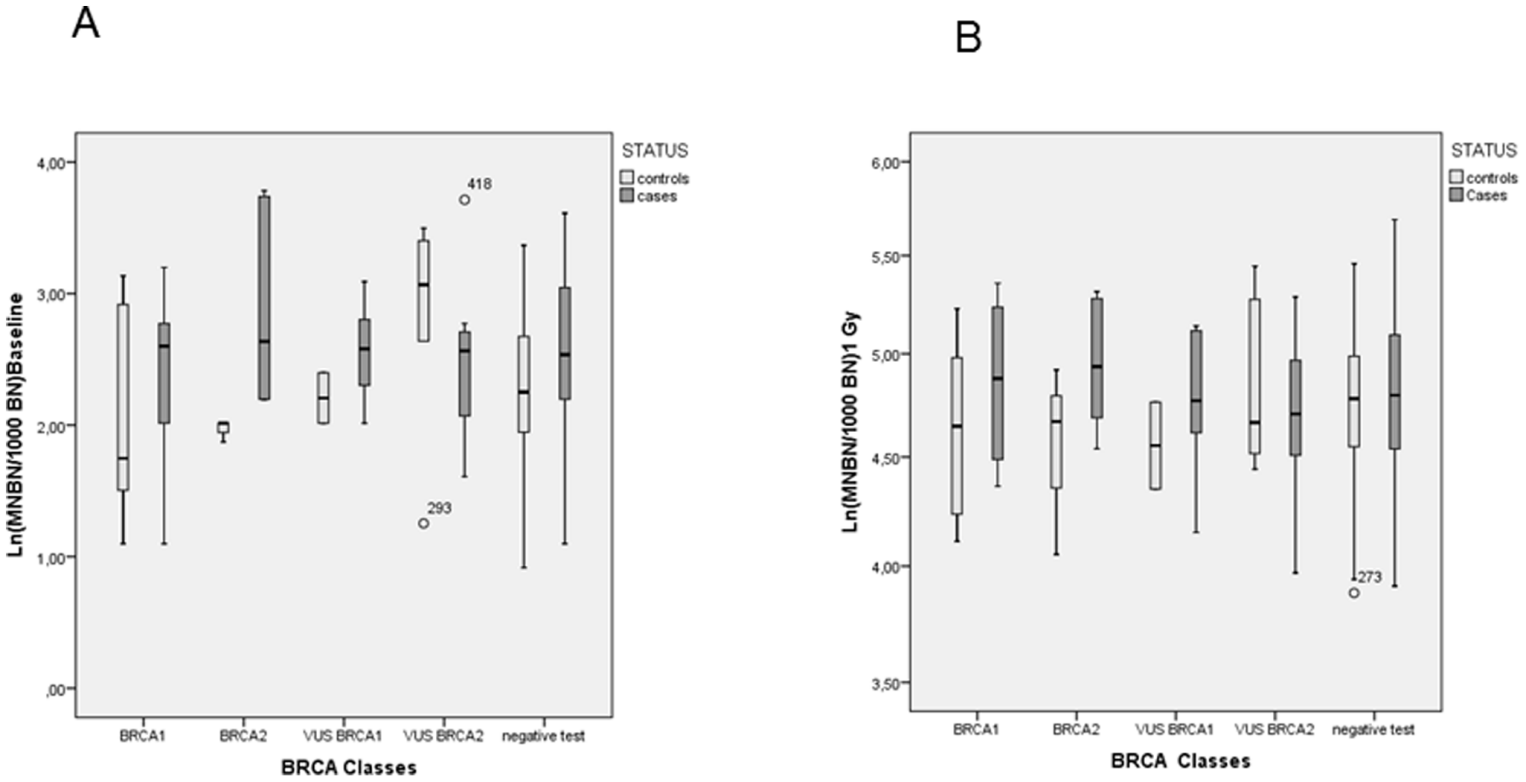
Box plot of frequency of Ln BNMN/1000 cells by BRCA status at baseline (panel A) and after in vitro irradiation (panel B). Box plots: the center horizontal line marks the median of the sample. The length of each box shows the range within which the central 50% of the values fall, with the top and bottom of the box at the first and third quartiles. The vertical T-lines represent intervals in which 90% of the values fall. The symbols show outliers.

Four BRCA classes were considered on the basis of the presence of a pathogenic and uncertain significance (VUS) variants of BRCA1 and BRCA2 genes. Again, no clear pattern of an increased MN frequency, either at baseline or after irradiation, was seen in association with the presence of BRCA mutations or VUS (Table 3 and Fig 2). However the highest mean MNBN/1000 BN lymphocytes at baseline and after irradiation was observed in BC cases with BRCA2 pathogenic variants.

Among controls, the highest mean value of baseline and after irradiation MN was observed in VUS BRCA2 variants.

### Detailed results of MN by BRCA classes and variants

A large interindividual variability was observed in the different BRCA classes not related to any specific variant (table S1) (the localization of variants identified in breast cancer patients and controls is reported in figure S1 A and B). Among the subjects carrying pathogenic BRCA2 variants, two outliers were identified with MNBN/1000 BN out of the normal range (42, 43 and 203, 195,9 at baseline and after irradiation respectively), explaining the highest mean MN value observed in BC, BRCA2-positive cases.

### Multivariate analyses

In multivariate analyses the logarithms of baseline MN frequency and of MN frequency after irradiation were modeled as a function of the following covariates: year of sample collection/analysis, age (in 4 groups), Menopausal status, Family History of breast Cancer, number of children (table 4). Baseline MN frequency was found to be associated only with year of sample collection/analysis and with age (p<0.0001 and p<0.0001, respectively). No association was found with the case/control status (p = 0.602) nor with a Family History of BC (p = 0.733). After irradiation, MN frequency was still significantly associated with year of sample collection/analysis (p<0.001), but the association with the age of the individual was no longer significant (p = 0.192). Furthermore, an association with the case/control status was seen (p = 0.012), with controls showing higher mean values. However, the size of the difference was minimal (0.067 on a log scale, corresponding to a proportional increase of 7%). Again, no association with BC FH was observed (p = 0.209).

**Table 4:** Multivariate Analysis of LnMN/1000 cells at baseline and after irradiation as function of years of analysis, age classes, menopausal status, family history of breast cancer.

Factor	Coefficient	S.E.	df	p-value	Coefficient	S.E.	df	p-value
	Ln MNBN/1000 cells baseline				Ln MNBN/1000 cells after irradiation			
Intercept	2.941	0.113	1	0.000	5.056	0.056	1	0.000
**Year of sample collection/analysis**			5	0.000			5	0.000
2006–7	−0.326	0.099			−0.682	0.049		
2008	−0.184	0.119			−0.297	0.059		
2009	−0.159	0.062			−0.172	0.030		
2010	−0.195	0.089			0.079	0.044		
2011	−0.232	0.080			−0.030	0.039		
2012	reference				reference			
**Age classes**			3	0.000			3	0.192
<40	−0.511	0.115			−0.112	0.057		
40–55	−0.184	0.087			−0.45	0.043		
55–65	0.061	0.083			−0.35	0.041		
>65	reference				reference			
**Menopausal status**			1	0.181			1	0.837
premenopausal	0.026	0.072			0.005	0.035		
postmenopausal	reference				reference			
**N. of children**			3	0.089			3	0.525
Nulliparous	−0.206	0.105			0.001	0.052		
1	−0.212	0.100			0.014	0.049		
2	−0.118	0.099			0.041	0.049		
>2	reference				reference			
**Family History of Breast Cancer**			3	0.733			3	0.209
1^st^ degree	0.010	0.060			−0.001	0.029		
2^nd^ degree	−0.008	0.065			0.024	0.032		
3^rd^ degree	0.061	0.120			0.112	0.059		
negative	reference				reference			
**Status**			1	0.602			1	0.012
controls	0.028	0.054			0.067	0.027		
cases	reference				reference			

## Discussion

The aim of the present study was to evaluate the potential role of micronucleus test in identifying a susceptibility to BC related to familiar or genetic factors. The MN frequency in peripheral lymphocytes at baseline level and after an in vitro challenge with ionizing radiations was compared in groups of healthy women and breast cancer patients with or without family history and in a subgroup of women evaluated for the presence of pathogenic BRCA1/2 mutations.

Overall, no strong or consistent association between MN frequency and BC status or family history and BRCA status was detected, neither at baseline nor after in vitro challenge. In particular, the primary study hypothesis of an increased radiation sensitivity of lymphocytes from women with a family history of BC or with a BRCA mutation, as assessed by the MN test, is not supported by our results. Among the determinants of MN frequency that were investigated in our study, only age was clearly related to an increase of MN frequency both at baseline and after in vitro irradiation, in line with the large majority of available studies [17]. No clear effect on the MN frequency of other variables, such as BMI, age at menarche, age of first pregnancy, number of children, smoking habits and oral contraceptive use was observed.

Our negative results apparently conflict with those of several previous studies that are often quoted in support of an association between BC risk [26]–[28], or inheritance [21]–[23] and MN frequency. However, most of these studies were carried out in small groups of cases with known or putative genetic predisposition to BC. Furthermore, when considered collectively, their results are inconsistent and do not allow to draw any clear conclusion [7]. MN frequency, both at baseline and after in vitro challenge, is higher in BC patients than in controls in the majority of these studies, but the BC family history was seldom, if ever, adequately addressed. Increase in MN frequency after in vitro irradiation, in association with the presence of BRCA1/2 pathogenic mutations, was observed only in small studies [21]–[23] but was not confirmed in the larger ones [29]–[31].

Our recent metanalysis [7], confirmed the presence of a rather consistent increase of baseline MN frequency in BC cases when compared to controls, albeit in the presence of a large inter-individual variability. However, no association with family history of BC nor with the presence of BRCA mutations was observed. The in vitro challenge with ionizing radiations was not associated with any improvement in the ability of MN test to discriminate between individuals with and without family history of BC or BRCA mutations.

Therefore, the results of the present study are not in contrast with those of the available literature [7], [32], and the reliability of its negative findings is supported by its size and its methodology. To the best of our knowledge this study, with 515 women, is the largest study ever carried out to evaluate the potential role of MN assay in BC. All recruited subjects were carefully characterized for BC family history by specialised personnel. Cancer patients were enrolled only if they had completed chemo- or radio-therapy at least by 12 months at the time of blood sampling. The blood samples were processed and analysed in the same lab using a standardized protocol and the MN scoring was performed by experienced intercalibrated scorers.

Yet, although we attempted to address most of the biases affecting previous studies, the possibility that our negative results are due to some uncontrolled confounding factors cannot be ruled out. First, although subject recruitment involved a careful control for the exposure to antiblastic drugs or radiations in cancer patients, the presence of exposures affecting the genotoxic response such as ionizing radiations for diagnostic purposes or drug intake cannot be excluded. For instance, mammography, even at low doses, produces clustered DNA damage that is difficult to repair and results in an increase in MN frequency [33].

Furthermore, our study lasted more than six years and we observed an association of the MN frequency at baseline and after irradiation with year of sample collection/analysis suggesting the involvement of unknown technical factors associated with the sample processing and scoring and/or a variability in the irradiation dose during the time.

To this regard, it must be underlined that adjustment for year of sample collection/analysis in multivariate analyses failed to produce any detectable change in the results of our analyses, as far as the association between MN frequency and BC family history of presence of BRCA mutations.

In addition, the use of a high irradiation rate in our study, which resulted in a very short exposure period, could have been a source of uncertainty in delivery of dose. Furthermore, the challenge dose selected for this study may not be the most appropriate to observe differences in MN frequency associated with DNA repair capacity, and further investigations are needed to explore the optimal radiation challenge dose, dose rate, and radiation quality required to obtain optimal discrimination in this kind of studies.

At any rate, these factors can only partially explain the large variability in the MN frequency evidenced at baseline and after the irradiation in all groups. This inter-individual variability is common to the large majority of studies involving the application of MN assay in patients with cancer [5], [6], or different degenerative diseases [11], [12] and suggests that confounding factors, apart from those already identified, could be involved in the induction of MN frequency, making it difficult to evaluate the presence of differences between different groups of individuals. Another possibility worth discussing is that the MN test, either at baseline or after irradiation, is not the appropriate test to evaluate the DNA repair deficiency and the consequent radiation sensitivity which have been postulated to cause the increased cancer risk of women with BRCA mutations.

The MN assay has been successfully applied in detecting increased chromosomal instability in different syndromes characterized by defective mutations in DNA repair genes [13], [14], [34], but it's not clear if the test is enough sensitive in revealing the effects of deficiencies in DNA repair cofactors, associated with BRCA1/2 mutations.

The mutagen sensitivity assay was developed in cultured peripheral lymphocytes by the application of chromosomal aberration test to reveal the chemical or radiosensitivity in cancer patients. Increased G2-phase chromosomal radiosensitivity in BC patients with high interindividual variability, evaluated as ionizing radiation-induced chromatid breaks, were described in early studies [27], [29], [35]–[36] and confirmed in a recent one [37]. However, no association between radiation sensitivity and family history or BRCA1/2 mutations was consistently observed [38], [39], providing indirect support for our results.

In conclusion, our results indicate that the MN assay in peripheral lymphocytes, as conducted in our study, cannot be considered a marker of BC risk or susceptibility.

Any future evaluation of a potential clinical role of MN assay as a biomarker in cancer or degenerative diseases, involves the identification of the unknown confounding factors that are responsible for the huge, largely unexplained, inter-, and possibly intra-individual variability. Further studies are needed to address this variability through repeated analyses in groups of healthy subjects, carefully controlled for individual characteristics and sampling conditions.

## Supporting Information

Figure S1 **Localization of variants identified in breast cancer patients and controls: BRCA1 protein.**
(TIF)

Figure S2 **Localization of variants identified in breast cancer patients and controls: BRCA 2 protein.**
(TIF)

Table S1 **Effect of BRCA 1 and 2 pathogenic variants on frequency of MNBN/1000 BN lymphocytes at baseline and after in vitro irradiation at 1 Gy.**
(DOC)
