# Supplementary figures and images for: Clinical Application of Micronucleus Test: A Case-Control Study on the Prediction of Breast Cancer Risk/Susceptibility
Source: PLoS One. 2014 Nov 21;9(11):e112354. doi: 10.1371/journal.pone.0112354 (PMC4240584; doi:10.1371/journal.pone.0112354)

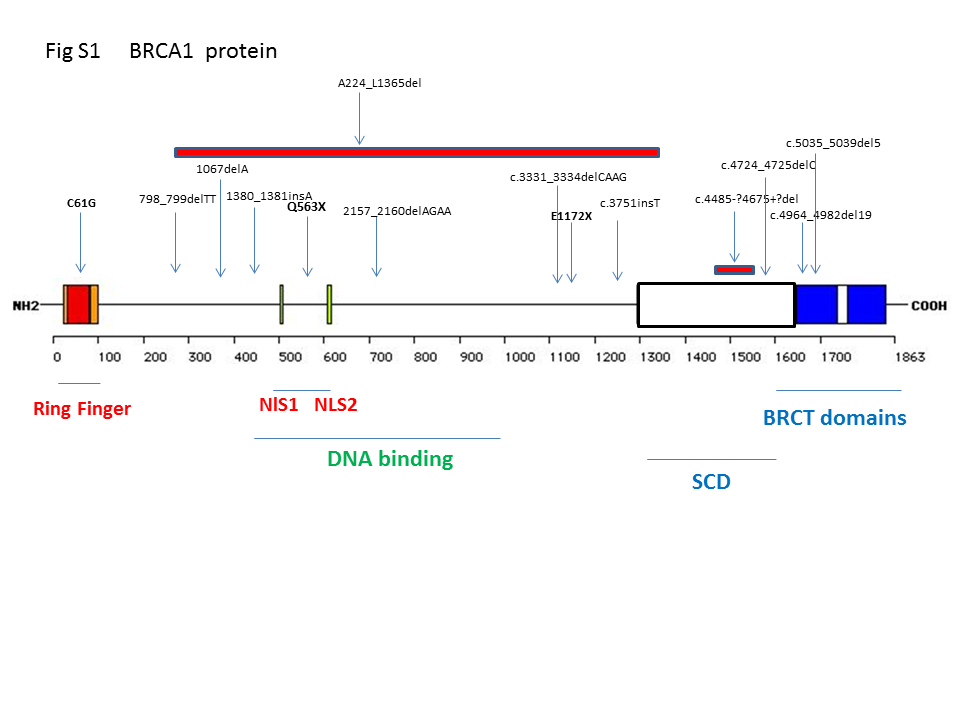

Supplement: Figure S1 — Localization of variants identified in breast cancer patients and controls: BRCA1 protein. (TIF) [file pone.0112354.s001.tif]

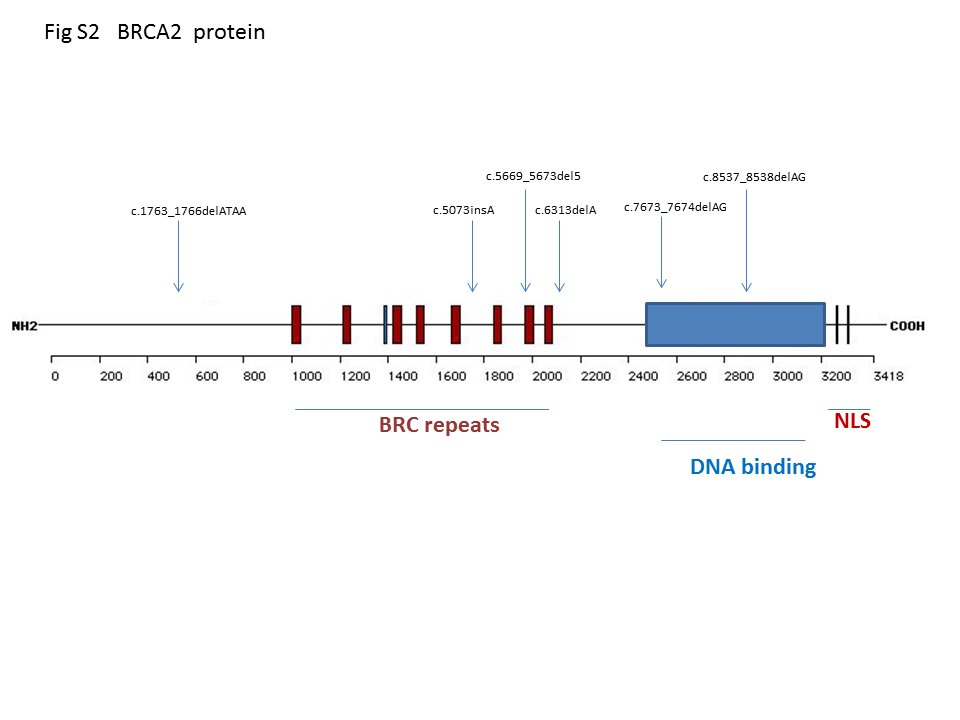

Supplement: Figure S2 — Localization of variants identified in breast cancer patients and controls: BRCA 2 protein. (TIF) [file pone.0112354.s002.tif]
