# Supplementary material for: Clinical Application of Micronucleus Test: A Case-Control Study on the Prediction of Breast Cancer Risk/Susceptibility
Source: PLoS One. 2014 Nov 21;9(11):e112354. doi: 10.1371/journal.pone.0112354 (PMC4240584; doi:10.1371/journal.pone.0112354)
Supplement: Table S1 — Effect of BRCA 1 and 2 pathogenic variants on frequency of MNBN/1000 BN lymphocytes at baseline and after in vitro irradiation at 1 Gy. (DOC) [file pone.0112354.s003.doc]

**Table S1** Effect of BRCA 1 and 2 pathogenic variants on frequency of MNBN/1000 BN lymphocytes at baseline and after in vitro irradiation at 1 Gy

| **Variants** | **N° of patients**  **Cases/Controls** | **First Variant** | | **MNBN/1000BN** | |
| --- | --- | --- | --- | --- | --- |
| **Pathogenetic**  **Variants BRCA1** |  | **HGVS: genomic level** | **HGVS: protein level** | **Baseline mean (S.E)** | **1 Gy mean (S.E.)** |
|  |  |  |  |  |  |
|  | 1/0 | c.181T>G | p.C61G | 12,5 | 124.5 |
|  | 1/0 | c.798_799delTT | p.S267fs | 15 | 78.5 |
|  | 0/2 | c.1067delA | p.Q356fs | 4 (1.00) | 61.1 (0.10) |
|  | 0/3 | c.1380_1381insA | p.F461fs | 6.67 (0.92) | 109.33 (16.49) |
|  | 1/0 | c.1687C>T | p.Q563X | 10 | 212 |
|  | 0/1 | c.2157_2160delAGAA | p.K719fs | 3,5 | 69 |
|  | 0/1 | c.3331_3334delCAAG | p.Q1111fs | 20 | 145.5 |
|  | 1/0 | c.3751insT | p.C1251fs | 3 | 82,5 |
|  | 1/0 | c.4096+1G>A | p.Ala224_Leu1365del | 7.5 | 91 |
|  | 1/0 | c.4485-?_4675+?del | p=? | 16 | 189 |
|  | 3/1 | c.4724_4725delC | p.P1575fs | 15.00 (4.00) | 137.81 (28.25) |
|  | 1/1 | c.4964_4982del19 | p.S1655fs | 10.00 (5.50) | 125.00 (13.00) |
|  | 2/0 | c.5030_5033delCTAA | p.T1677fs | 18.25 (3.75) | 147.05 |
|  | 0/1 | c.5035_5039del5 | p.L1679fs | 23 | 166 |
|  | 2/0 | c.3514G>T | p.E1172X | 15.5 (6.50) | 180.00 (9.00) |
| **BRCA2** |  |  |  |  |  |
|  | 1/0 | c.1763_1766delATAA | p.N588fs | 42 | 203 |
|  | 1/0 | c.5073insA | p.W1692fs | 8.96 | 114.5 |
|  | 1/0 | c.5669_5673del5 | p.M1890fs | 9 | 93.57 |
|  | 1/0 | c.6313delA | p.I2105fs | 9.5 | 108.5 |
|  | 1/0 | c.7673_7674delAG | p.E2558fs | 43.89 | 195.93 |
|  | 1/3 | c.8537_8538delAG | p.E2846fs | 10.50 (3.34) | 117.75 (23.90) |
| **VUS variants** |  |  |  |  |  |
| **BRCA1** |  |  |  |  |  |
|  | 1/0 | c.734A>T | p.Asp245Val | 10 | 124 |
|  | 1/0 | c.1934C>A | p.S645Y | 12 | 170.5 |
|  | 1/0 | c.2634A>G | p.A878A | 14.5 | 112 |
|  | 0/1 | c.2885A>G | p.E962G | 11 | 117 |
|  | 1/0 | c.4009G>C | p.D1337H | 16.5 | 166.5 |
|  | 1/0 | c.4484+65G>A | p=? | 7.5 | 63.5 |
|  | 1/0 | c.5074+12A>T | p=? | 22 | 101 |
|  | 0/1 | c.5509T>C | p.W1837R | 7.5 | 77.5 |
| **BRCA2** |  |  |  |  |  |
|  | 0/1 | c.-259G>C | p=? | 33 | 231.5 |
|  | 1/0 | c.-39delTCT | p=? | 5 | 96.5 |
|  | 2/0 | c.68-7T>A | p=? | 28.5 (12.50) | 141.00 (26.00) |
|  | 0/1 | c.316+5G>A | p=? | 14 | 85 |
|  | 1/0 | c.682-32A>G | p=? | 7 | 91.5 |
|  | 1/0 | c.3392G>A | p.R1131K | 7 | 197.5 |
|  | 1/0 | c.3885A>G | p.Q1295Q | 9 | 90 |
|  | 1/0 | c.4068G>A | p.L1356L | 13 | 156 |
|  | 0/1 | c.5423T>C | p.I1808T | 3,5 | 106 |
|  | 1/0 | c.6014A>G | p.D2005G | 15.48 | 132.27 |
|  | 1/0 | c.7435+54G>A | p=? | 13 | 53 |
|  | 1/0 | c.7505G>A | p.R2502H | 30 | 195 |
|  | 0/1 | c.7505G>A | p.R2502H | 13.5 | 110.,5 |
|  | 0/1 | c.8830A>T | p.I2944F | 21.5 | 91.5 |
|  | 1/0 | c.9648+42A>G | p=? | 14.5 | 74.5 |

S.E. standard errror
